# Transient mRNA CAR T cells targeting GD2 provide dose-adjusted efficacy against diffuse midline glioma and high-grade glioma models

**DOI:** 10.1093/neuonc/noaf115

**Published:** 2025-05-24

**Authors:** Jessica B Foster, Peter J Madsen, Kyra Harvey, Crystal Griffin, Allison Stern, Luke Patterson, Nikhil Joshi, Conor Dickson, Olivia McManus, Ezra Beaubien, Cullen Wilson, David R Beale, Valerie Baubet, Peeyush N Goel, Nicholas A Vitanza, Javad Nazarian, Mateusz Koptyra, Phillip B Storm, Adam C Resnick

**Affiliations:** Department of Pediatrics, Perelman School of Medicine, University of Pennsylvania, Philadelphia, PA, USA; Division of Oncology, Children’s Hospital of Philadelphia, Philadelphia, PA, USA; Center for Data Driven Discovery in Biomedicine, Children’s Hospital of Philadelphia, Philadelphia, PA, USA; Division of Neurosurgery, Children’s Hospital of Philadelphia, Philadelphia, PA, USA; Division of Oncology, Children’s Hospital of Philadelphia, Philadelphia, PA, USA; Division of Oncology, Children’s Hospital of Philadelphia, Philadelphia, PA, USA; Division of Oncology, Children’s Hospital of Philadelphia, Philadelphia, PA, USA; Division of Oncology, Children’s Hospital of Philadelphia, Philadelphia, PA, USA; Division of Oncology, Children’s Hospital of Philadelphia, Philadelphia, PA, USA; Center for Data Driven Discovery in Biomedicine, Children’s Hospital of Philadelphia, Philadelphia, PA, USA; Division of Oncology, Children’s Hospital of Philadelphia, Philadelphia, PA, USA; Division of Oncology, Children’s Hospital of Philadelphia, Philadelphia, PA, USA; Center for Data Driven Discovery in Biomedicine, Children’s Hospital of Philadelphia, Philadelphia, PA, USA; Center for Data Driven Discovery in Biomedicine, Children’s Hospital of Philadelphia, Philadelphia, PA, USA; Center for Data Driven Discovery in Biomedicine, Children’s Hospital of Philadelphia, Philadelphia, PA, USA; Center for Data Driven Discovery in Biomedicine, Children’s Hospital of Philadelphia, Philadelphia, PA, USA; Department of Pediatrics, Seattle Children’s Hospital, University of Washington, Seattle, WA, USA; Ben Towne Center for Childhood Cancer and Blood Disorder Research, Seattle Children’s Research Institute, Seattle, WA, USA; Department of Pediatrics, University Children’s Hospital, University of Zurich, Zurich, Switzerland; Center for Data Driven Discovery in Biomedicine, Children’s Hospital of Philadelphia, Philadelphia, PA, USA; Center for Data Driven Discovery in Biomedicine, Children’s Hospital of Philadelphia, Philadelphia, PA, USA; Division of Neurosurgery, Children’s Hospital of Philadelphia, Philadelphia, PA, USA; Center for Data Driven Discovery in Biomedicine, Children’s Hospital of Philadelphia, Philadelphia, PA, USA; Division of Neurosurgery, Children’s Hospital of Philadelphia, Philadelphia, PA, USA

**Keywords:** brain tumor, CAR T cells, diffuse midline glioma, mRNA, pediatric

## Abstract

**Background:**

Diffuse midline glioma (DMG) and high-grade glioma are devastating pediatric central nervous system tumors that remain incurable. Recent chimeric antigen receptor (CAR) T cell studies have shown proof of concept and early signs of efficacy against DMG targeting GD2. Prior work and ongoing clinical trials have focused on using viral vectors to create permanent CAR T cells. However, virally transduced GD2-directed CAR T cells have shown significant neurotoxicity in both preclinical models and human trials.

**Methods:**

We evaluated transient CAR T cells targeting GD2 created with mRNA, assessing for efficacy and safety in cell line, organoid, and in vivo xenograft models with repetitive intratumoral dosing.

**Results:**

We show that mRNA GD2-directed CAR T cells are active against both cell lines and organoid models of DMG and high-grade glioma in vitro. Cytotoxicity consistently abates over 9 days, highlighting the potential to avoid toxicity from persistent T cell activity. In both pontine and thalamic DMG xenograft models, repeated doses of mRNA GD2-directed CAR T cells were titrated down to maintain therapeutic effects without causing neurologic toxicity.

**Conclusions:**

Our results demonstrate the utility of transient mRNA CAR T cells delivered intratumorally to provide effective tumor killing with a defined half-life, allowing for modulation of the dose and potential side effects. We anticipate this study will expand the use of CAR T cell therapy for DMG and other central nervous system tumors and non-malignant disorders, where concern for toxicity from permanently expressing CAR T cells may hinder development.

**Key Points**

• mRNA CAR T cells can safely target GD2 in DMG models without neurotoxicity
• Targeting GD2 may also be an effective strategy in other high-grade gliomas
• Transient mRNA CAR T cells offer the flexibility to titrate doses up and down and finetune the effect of CAR therapy in the brain

**Importance of the Study**

Diffuse midline glioma remains an incurable tumor. CAR T cells have shown signs of efficacy for patients in ongoing trials, but benefit has been inconsistent, and targeting GD2 has resulted in neurotoxicity. Transient mRNA CAR T cells offer an alternative strategy, with the benefit of increased flexibility in dosing to allow for titration. This strategy can be used for controlled CAR T cell activity in the brain, opening the possibility of treating all patients regardless of tumor location or size.

Diffuse midline glioma (DMG) is a devastating pediatric central nervous system (CNS) tumor with no known curative therapy and a median survival of around 1 year.^1,2^ This subset of pediatric high-grade glioma (pHGG) occurs in the midline, most often the pons or thalamus, and is defined by histone H3K27 alterations, either through the characteristic H3K27M mutation or EZHIP overexpression.^3^ Evaluations of DMG biology identified that the cell surface disialoganglioside GD2 is highly expressed in DMG, making it an attractive immunotherapeutic target.^4^

Chimeric antigen receptor (CAR) T cell therapy has been a successful immunotherapy strategy for leukemia, lymphoma, and multiple myeloma, with 6 FDA-approved products.^5^ Success in solid and CNS tumors has been slower, with many failed studies but recent early clinical trial results showing promise.^6–11^ Initial work using GD2-directed CAR T cells for DMG has utilized conventional lentiviral vectors to create permanently transduced CAR T cells.^4,8,10^ While viral vectors provide persistent CAR expression, these GD2-directed CAR T cells also have been associated with toxicity from localized inflammation.^4,12^ Tumors in the midline are particularly at risk for severe neurologic complications secondary to inflammation, such as fatal obstructive hydrocephalus and/or herniation. In their preclinical modeling, Mount et al showed mice with DMG xenografts in the thalamus could not tolerate GD2-directed CAR T cell therapy due to fatal herniation,^4^ prompting the initial exclusion of DMG patients with thalamic or cerebellar disease from the subsequent clinical trial (NCT04196413).^10^ Given that many patients will either present with primary thalamic tumor or develop an extension of their pontine tumor into the thalamus or cerebellum, we sought to explore alternative treatment strategies with GD2-directed CAR T cells that would maintain safety for all DMG patients.

Building on our prior work showing effective CAR T cells can be created using electroporation of mRNA,^13^ we hypothesized that the transient nature of mRNA CAR T cells could be exploited to titrate the dose and degree of inflammation in DMG tumors to allow for safe and efficacious treatment. In prior studies, systemic delivery of transient mRNA CAR T cells has not been sufficient to provide long-term disease control in solid tumor models,^14,15^ however local and regional delivery into solid tumors has been shown to mediate antitumor activity,^14–18^ including our prior work using mRNA GPC2-directed CAR T cells in pediatric CNS tumor xenografts.^19^ In the current study, we take advantage of GD2 overexpression in DMG, using serial intratumoral delivery of GD2-directed mRNA CAR T cells as a therapeutic strategy.

## Methods

### Tumor Cell Lines and Organoids

Pediatric CNS cell tumor cell lines were provided by the Children’s Brain Tumor Network (CBTN), or the generous gifts of Michele Monje (Stanford University) and Sabine Mueller (University of San Francisco, California). Cell lines were maintained in culture as previously described^19^ in serum-free conditions. GFP and luciferase were introduced into the cell lines as previously described,^19^ using either standard transduction of lentiviral plasmid pLL-EF1a-rFLuc-T2A-GFP-mPGK-Puro (System Biosciences) or pre-made lentivirus (Cellomics). All cell lines were confirmed with STR testing and negative for mycoplasma.

Organoids were created from fresh human DMG tumors by the CBTN. Briefly, fresh tumor material was roughly cut into 0.5 cm size pieces and maintained under constant shaking conditions in serum-free media. Following propagation, organoids were cryogenically frozen prior to use in cytotoxicity experiments.

### CAR T Cell Generation

Second-generation CAR T cells with 14G2a GD2-binding domain and 4-1BB and CD3 zeta co-stimulatory domains were used in this study.^4^ Plasmid encoding the CAR in 1658 vector, which is optimized for mRNA production (gift of Katalin Kariko), was synthesized by Genscript. mRNA was produced *in vitro* using MEGAscript T7 transcription kit (Invitrogen), followed by capping (ScriptCap m7G Capping System and ScriptCap 2’-O-Methyltransferase Kit, Cellscript) and poly-A tailing (Poly(A) Tailing Kit, Invitrogen) as previously described,^13^ or manufactured by the University of Pennsylvania mRNA Core, including incorporation of N-methylpseudouridine and purification to remove dsRNA.

mRNA CAR T cells were generated using mRNA as previously described.^13,14^ Briefly, healthy human T cells were obtained from the University of Pennsylvania Immunology Core then activated and expanded on Dynabeads CD3/28 beads (Gibco) and cryogenically frozen. At the time of use, T cells were thawed and rested for 12 to 24 h, washed 3 times in Optimem, and then transfected with mRNA at a ratio of 1μg of mRNA per 1 × 10^6^ T cells using the ECM 830 (BTX) electroporation systems at 500 V and 700 to 800 μs. T cells rested for 24 hours following transfection and CAR expression was confirmed with flow cytometry immediately prior to use.

### GD2 and CAR Flow Cytometry

GD2 expression was analyzed on tumor cell lines using PE Mouse Anti-Human Disialoganglioside GD2 (BD Biosciences, Cat no. 562100). Flow cytometry to confirm GD2 CAR expression was completed using biotin-SP-AffiniPure Goat Anti-Mouse IgG F(ab’)2 Fragment Specific (Jackson ImmunoResearch Laboratories, Inc. Cat no. 115-065-072), with PE-Streptavidin secondary (BD Pharmingen, Cat no. 554061) as previously described.^20^ CD19 CAR controls were confirmed using Biotin-protein L (GenScript, Cat no. M00097) as previously described.^13^ Flow cytometry was completed on Accuri C6 (BD) or CytoFlex (Beckman-Coulter). Analysis was completed using FlowJo v10 (BD).

### RNA Sequencing

RNA sequencing of 395 pediatric primary high-grade brain tumor specimens have been previously generated by the CBTN and accessed from OpenPBTA v12.^21^ RNA sequencing of 2642 adult normal brain samples have been generated and made available on the Genotype-Tissue Exploration (GTEx) Portal.^22^ RNA sequencing analysis was performed in R 4.3.1. Gene count data for each sample was logarithmically transformed and normalized before pairwise comparisons between cohorts. The R/Bioconductor software limma^23^ was used to complete differential gene expression analysis with an empirical Bayes moderated *t*-test and the Benjamini–Hochberg correction performed for P-value adjustment.

### mRNA Frameshift Evaluation

Putative N-methylpseudouridine induced + 1 ribosome frameshifting products^24^ can potentially lead to additional transcription sequences, with subsequent off-target effects of mRNA-based therapeutics. To ensure there were no off-target effects, we screened for ribosomal slippery sequences that could potentially translate into frameshifting-induced protein products. We established a computational pipeline in R identifying all in-frame mRNA slippery sequences with UUUX or AAAG, where X = U or C, and altered these sequences to remove X/G from mRNA sequences. Altered mRNA sequences were then run through BLASTx^25^ to confirm the prevention of cross-reactive mRNA sequences induced by possible + 1 ribosomal frameshifting.

### Penalized Regression Modeling for Variable Selection

To identify relationships between GD2 Enzyme expression and mutational characteristics, we ran numerous penalized linear regression models. We integrated harmonized linearized copy-number alteration data, mutational data, and transcripts-per-million RNA expression data from DMG samples abstracted from the OpenPBTA v12. Taken together, we applied least absolute shrinkage and selection operator (LASSO) linear regression, ridge linear regression, and elastic-net linear regression models to identify mutational features that could predict for GD2 enzyme expression. The dependent variable in our supervised model was set to be the mean transcripts-per-million expression of B4GALNT1, ST8SIA1, and ST3GAL5 for each sample. We applied each model using 10-fold cross-validation. Alpha values for penalization optimization of elastic-net regression were optimized by screening across a variety of values between 0 and 1 and selecting the alpha value that minimized the cross-validation error. All analyses were completed in R 4.3.1 utilizing the package glmnet.^26^

### In Vitro *Cytotoxicity Assessment*

CAR T cell efficacy was assessed in vitro using xCELLigence Real-Time Cell Analysis system (RTCA, Agilent) for adherent cell lines, and Bright-Glo luciferase assay system (Promega) or Incucyte Live-Cell Analysis (Sartorius) for suspension cell lines, as previously described.^19^ Briefly, tumor cells were plated at 50,000 cells per well and allowed to rest for 24 hours. CAR T cells were then plated at varying E:T ratios in triplicate. RTCA and Incucyte plates were monitored for 5 days. Luciferase assays were assessed at 48 hours, and 100% cytotoxicity was determined by tumor cell treated with 10% triton as a control.

For patient-derived organoid cytotoxicity assay, organoids of estimated 0.4 to 0.6 mm size were selected (estimated 2 × 10^5^ cells/organoid), plated in triplicate in a 96 well plate. GD2-directed or CD19-directed CAR T cells were added with 5 × 10^5^ cells per well/per organoid, (2.5:1 E:T ratio) and incubated for 24, 48, and 72h. Organoids were washed, fixed and preserved for immunofluorescence (IF) analysis or dissociated into single-cell suspension followed by immunostaining and flow cytometry analysis.

### Immunofluorescence

The organoids were fixed in 4% PFA and embedded into cryomolds with tissue freezing medium (GeneralData, TFM-C) and flash frozen on powdered dry ice. 12 μm organoid sections were cut using a cryostat and melted onto charged slides (VWR, 48311-703). Cut slides were dried for 1 hour at room temperature. Sections were rehydrated and washed with Tris-Buffered Saline/ 0.1%Tween20 (TBST), blocked for an 1 hour at room temperature in a blocking buffer 10% donkey serum (Sigma) solution, 0.5% Triton X-100 (Sigma), 1% BSA (Sigma), 0.1% gelatin (Sigma), and 22.52 mg/ml glycine (Sigma) in TBST. Sections were incubated overnight at 4 °C with primary antibodies cleaved caspase-3 (CC3; Cell Signaling) and anti-human CD3 (BioLegend) in a TBST solution with 5% donkey serum and 0.1% Triton X-100, washed and incubated with secondary antibodies anti-mouse Alexa Fluor 488 (ThermoFisher), anti-rabbit Alexa Fluor 568 (ThermoFisher), and nucleus dye Hoechst 33342 (Sigma). The TrueBlack Lipofuscin (Biotium) was used for autofluorescence quenching, followed by mounting with Vectashield antifade mounting medium (Vector). Images were taken with Leica SP8 confocal microscope (Leica) with a white light laser to capture 2X, 10X, and 40X Z-stack images of entire organoid sections. Laser emission and gain settings were kept constant between treatments for quantitative image analysis.

The IF images were analyzed using CellProfiler 4.2.5.^27^ Multiplexed 40X and 2X images were input and thresholded using a minimum-cross entropy method and cell counts were identified utilizing staining intensity to distinguish distinct cells. Per-cell CC3 staining intensity was identified and cells with staining intensity greater than 2 standard deviations from the mean were omitted to account for noise.

### Organoid Dissociation and Flow Cytometry

The organoids were dissociated, and cells were washed and stained followed by analysis by flow cytometry. Briefly, for each time point, 3 organoids were resuspended in 1ml of prewarmed Trypsin (ThermoFisher) and incubated at 37° degrees for 15 minutes with intermittent pipetting. Trypsin was inactivated with 1 ml DMEM/F12 (Sigma) media with 10% FBS (HyClone), cells were washed with phosphate-buffered saline (PBS; Gibco) and immediately incubated with antibodies for 1 hour in room temperature. Samples were washed, resuspended in PBS with SYTOX® Red (Thermo) at 5 nM, and analyzed using flow cytometer CytoFLex (Beckman-Coulter).

### Xenografts

All mouse studies were completed under an approved Institutional Animal Care and Use Committee protocol at the Children’s Hospital of Philadelphia (IAC 19-000907). NOD-SCID-γc–/– (NSG) mice were purchased from Jackson or bred in-house and kept in pathogen-free conditions. Tumor cell lines containing luciferase were stereotactically injected into the pons (coordinates from lambda: A/P − 0.80 mm, D/V 5.00 mm, M/L − 1.00 mm) or thalamus (coordinates from bregma: A/P − 1.00 mm, D/V 3.50 mm, M/L − 0.80 mm) as previously described.^19,28^ Following tumor engraftment, CAR T cells were delivered intratumorally using indwelling cannulas as previously described.^29^

### In Vivo *Assessments*

Tumor size was monitored over time using bioluminescent imaging with In Vivo Imaging System (IVIS) Spectrum (Perkin Elmer) for DMG models 7316-6349 and SF8628. Models SUDIPG13P* and 7316-3058 do not maintain luciferase expression in vivo over time, and mice were monitored for overall survival as the primary endpoint. At the development of graft-versus-host disease (GVHD) or tumor endpoints, mice were euthanized and tumors were excised for immunofluorescence analysis.

### Statistical Analysis

Data was analyzed using GraphPad Prism v9 (GraphPad) and R 4.3.1. For flow cytometry, in vitro cytotoxicity, IF, and tumor burden by bioluminescent imaging, a comparison of means was completed using a Student *t*-test or 2-way analysis of variance. The overall survival of mice was analyzed using Kaplan–Meier curve and log-rank test. RNA sequencing differential gene expression analysis was completed with an empirical Bayes moderated *t*-test and the Benjamini–Hochberg correction performed for P-value adjustment. Data are presented as the mean with standard deviation, or as noted in results or figure legend. Number of samples, mice, and replicates for each experiment is also indicated in the results and figures.

## Results

### GD2 Is Expressed in Histone Mutant and Histone Wild-Type Pediatric High-Grade Glioma

**Figure 1.**
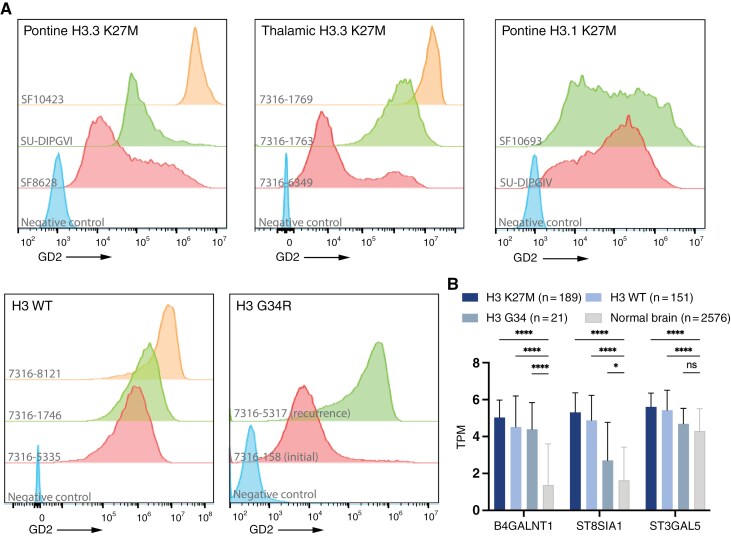
GD2 is expressed across histone mutant and wild-type pediatric high-grade gliomas. (A) Representative flow cytometry from H3.3 K27M, H3.1 K27M, H3G34R, and histone wild-type cell lines for GD2 surface expression. Graphs display fluorescence intensity. Cell lines indicated to the left on each row, and tumor type noted at the top of each box. GD2 negative control cell line shown at bottom in blue. (B) RNA sequencing of *B4GALNT*, *ST8SIA1* and *ST3GAL5* genes from histone variant primary tumor samples. Gene expression shown as mean TPM, error bars indicate SD. ****= *P* < .0001, * = *P* < .05, ns = not significant.

We first set out to verify GD2 expression in DMG cell line models, including several that are used for xenograft generation. Using flow cytometry, we confirmed high expression of GD2 in H3 mutant cell lines (Figure 1A, Supplementary Figure S1), as previously reported. Both pontine and thalamic H3.3 mutant cell lines were uniformly positive although with variable peak mean fluorescent intensity (MFI). Among the two H3.1 mutant cell lines tested, both also showed expression of GD2, but with more variable broad expression rather than a discrete peak compared to H3.3 samples, mean MFI 359711 for H3.1 versus 3771196 for H3.3 (*P* = n.s.) (Figure 1A). In addition, we tested H3 G34R mutant pHGG paired cell lines derived from the same patient, 1 from primary resection (7316-158) and the second at the time of recurrence (7316-5317) (Figure 1A). Both showed GD2 surface expression, however, the recurrent sample showed higher GD2 expression (MFI 117693 versus 793000, *P* < .0001) from this single patient. Finally, we also tested several histone wild-type (H3-WT) pHGG cell lines (Figure 1A). While previous work has suggested histone mutation drives GD2 expression,^4^ we also saw elevated GD2 expression in H3-WT pHGG cell lines, at levels similar to H3.3 mutant tumor cell lines, similar to other groups that have evaluated GD2 expression in histone wild-type gliomas at a protein level.^30,31^ To verify this observation, we also looked at IF of histone mutant and H3-WT pHGG cell lines for GD2 (Supplementary Figure S2). Both histone mutant and H3-WT showed strong GD2 cell surface staining, potentially extending this therapeutic target to all pHGG. Evaluation of RNA sequencing was in line with protein expression data, where we observed increase expression of enzymes upstream of GD2 production including *B4GALNT1*, *ST8SIA1*, and *ST3GAL5* for both H3 mutant and H3-WT tumors, compared to normal brain (Figure 1B). Of note, correlation of GD2 enzyme biomarkers utilizing somatic copy-number and mutation data alongside transcriptomic data yielded no significant relationships between GD2 enzyme expression and molecular profiles (Supplementary Figure S3).

### *mRNA GD2 CAR T Cells Show Transient But Potent Tumor Cell Cytotoxicity* In Vitro

**Figure 2.**
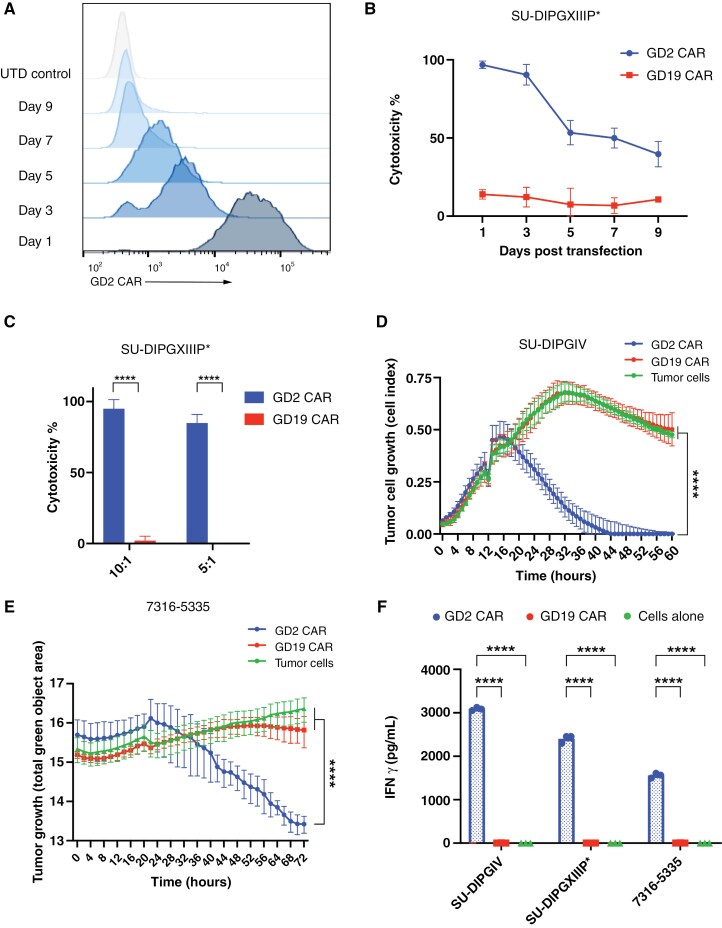
mRNA GD2 CAR expression and cytotoxicity in DMG and pHGG cell lines. (A) CAR expression over time following mRNA electroporation. Representative graphs of CAR expression from T cells electroporated with GD2-directed CAR mRNA in blue, showing modal fluorescence intensity over time. Stained untransfected T cell controls shown in light gray at the top as a negative control. UTD = untransfected. (B) DMG model SU-DIPGXIIIP* cytotoxicity with mRNA GD2-directed CAR T cells over time. CAR T cells were rested as indicated by days post-transfection prior to plating with tumor cells at E:T ratio 10:1. Cytotoxicity measured by luminescence after 48 hours of co-incubation. CD19-directed CAR T cells shown as negative control. (C) SU-DIPGXIIIP* tumor cells plated at 10:1 and 5:1 E:T ratios with mRNA CAR T cells, either GD2-directed CAR T cells or CD19-directed negative control CAR T cells. Cytotoxicity measured by luminescence after 48 hours of co-incubation. (D) SU-DIPGIV tumor cells plated on RTCA, with mRNA CAR T cells added at approximately 12 hours at E:T ratio 1:1. Tumor growth measured over time for tumor cells treated with GD2-directed CAR T cells, negative control CD19-directed CAR T cells, and vehicle only. (E) 7316-5335 plated on Incucyte, with mRNA CAR T cells added at approximately 24 hours at E:T ratio 5:1. Tumor growth measured over time for tumor cells treated with GD2-directed CAR T cells, negative control CD19-directed CAR T cells, and vehicle only. (F) IFN gamma measured by ELISA assay from experiments shown in C, D, and E. Supernatant was collected after 24 hours of co-incubation of tumor cells plated with mRNA CAR T cells and analyzed with ELISA. All graphs in B through F displayed as mean with SD. **** = *P* < .0001.

We next sought to test our transient mRNA GD2 CAR T cell constructs against DMG and pHGG cells. We electroporated mRNA encoding the GD2-directed CAR to create CAR T cells. Given recent reports of mRNA frameshifting from N1-methylpseudouridine,^24^ which is included in our mRNA transcripts, we created a tool to identify alternative open reading frames (ORFs) and evaluate for other protein products that may be produced. Our GD2-directed CAR mRNA sequence resulted in 2 potential frameshift sequences, both of which did not result in a functional protein sequence (Supplementary Table S1), suggesting no potential untoward effect on the T cells. Electroporated T cells showed over 90% CAR positivity on day 1 that degraded over time with minimal CAR detected by day 9 (Figure 2A). In parallel, CAR T cell activity decreased over time with maximal cytotoxicity at day 1 following transfection (Figure 2B). mRNA CAR T cells showed potent tumor-directed cytotoxicity in vitro against H3.3 (Figure 2C, Supplementary Figure S4) and H3.1 K27M mutant cell line models (Figure 2D), as well as H3-WT cell lines (Figure 2E). In addition, CAR T cells showed degranulation of IFN gamma in the presence of tumor cell lines compared to control CAR T cells (*P* < .0001) (Figure 2F). Following mRNA degradation over 9 days, CAR T cells showed minimal cytotoxicity (Figure 2B), highlighting their transient activity.

**Figure 3.**
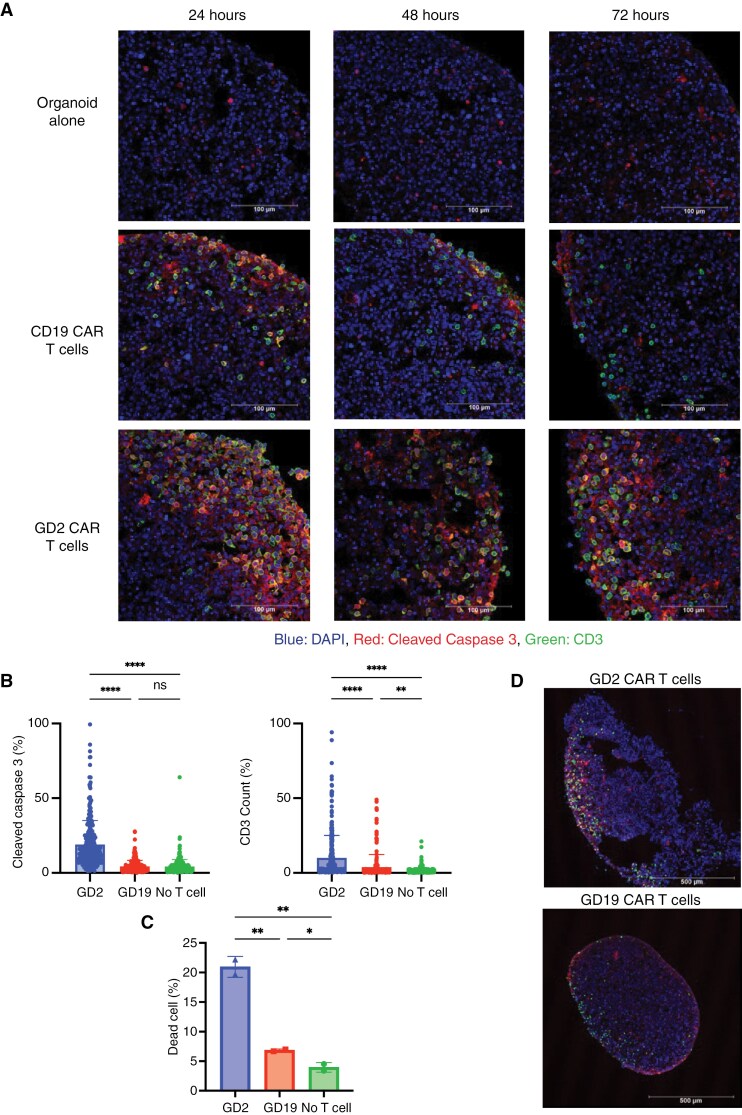
mRNA GD2 CAR T cell cytotoxicity in an organoid model of DMG. (A) Representative IF images of organoids with and without CAR T cells over 72 hours. Following co-culture with T cells, organoids were preserved and embedded at 24, 48, and 72 hours. Row displays each treatment group, columns indicate time points. Images acquired at 40x, scale bar is 100um. Colors indicated in legend at the bottom. (B) Quantification of CC3 positive cells (left) and CD3 T cells (right) at 72 hours across organoids with CAR T cells. Red (CC3) and green (CD3) cells were counted using CellProfiler. Individual counts for each section indicated by dots, and bar graph shows mean with SD. (C) Flow cytometry performed on single cell dissociated organoids, measuring percent of dead cells at 72 hours. Two replicates indicated by individual dots show the mean percentage of dead cells measured using SYTOX red. Bar graph displays mean across replicates with SD. (D) Representative IF images of organoids treated with CAR T cells at 10x magnification at 72 hours. GD2 CAR T cells in the top image, negative control CD19 CAR T cells in the bottom image. Scale bar is 500um. * = *P* < .05, ** = *P* < .01, **** = *P* < .0001.

We then tested our mRNA CAR T cells against an organoid model, generated from an H3.3 K27M mutant primary tumor sample that was minimally manipulated. Compared to organoids alone and CD19 CAR T cell control-treated organoids, we saw a significant increase in CD3 infiltration and apoptosis labeled by cleaved caspase 3 (CC3) for GD2 CAR T cell-treated organoids over 72 hours of co-incubation (CC3 mean area coverage 19.01% versus 4.509%, *P* < .0001 for GD2 versus CD19) (Figure 3A and B). Flow cytometry of dissociated organoids at 72 hours revealed higher cytotoxicity in GD2 CAR treated organoids, with 20.95% dead cells compared to 6.84% and 3.95% in CD19 treated and organoid alone controls, respectively (*P* < .01) (Figure 3C). Immunofluorescence of the organoids imaged at 10x power at 72 hours also revealed that GD2 CAR T cell-treated organoids qualitatively were no longer maintained whole and had deteriorated to multiple pieces, compared to CD19 CAR T cell-treated organoids which remained intact (Figure 3D).

### *Repeated Dosing of mRNA GD2-Directed CAR T Cells Show Efficacy in Pontine DMG* In Vivo *Models*

**Figure 4.**
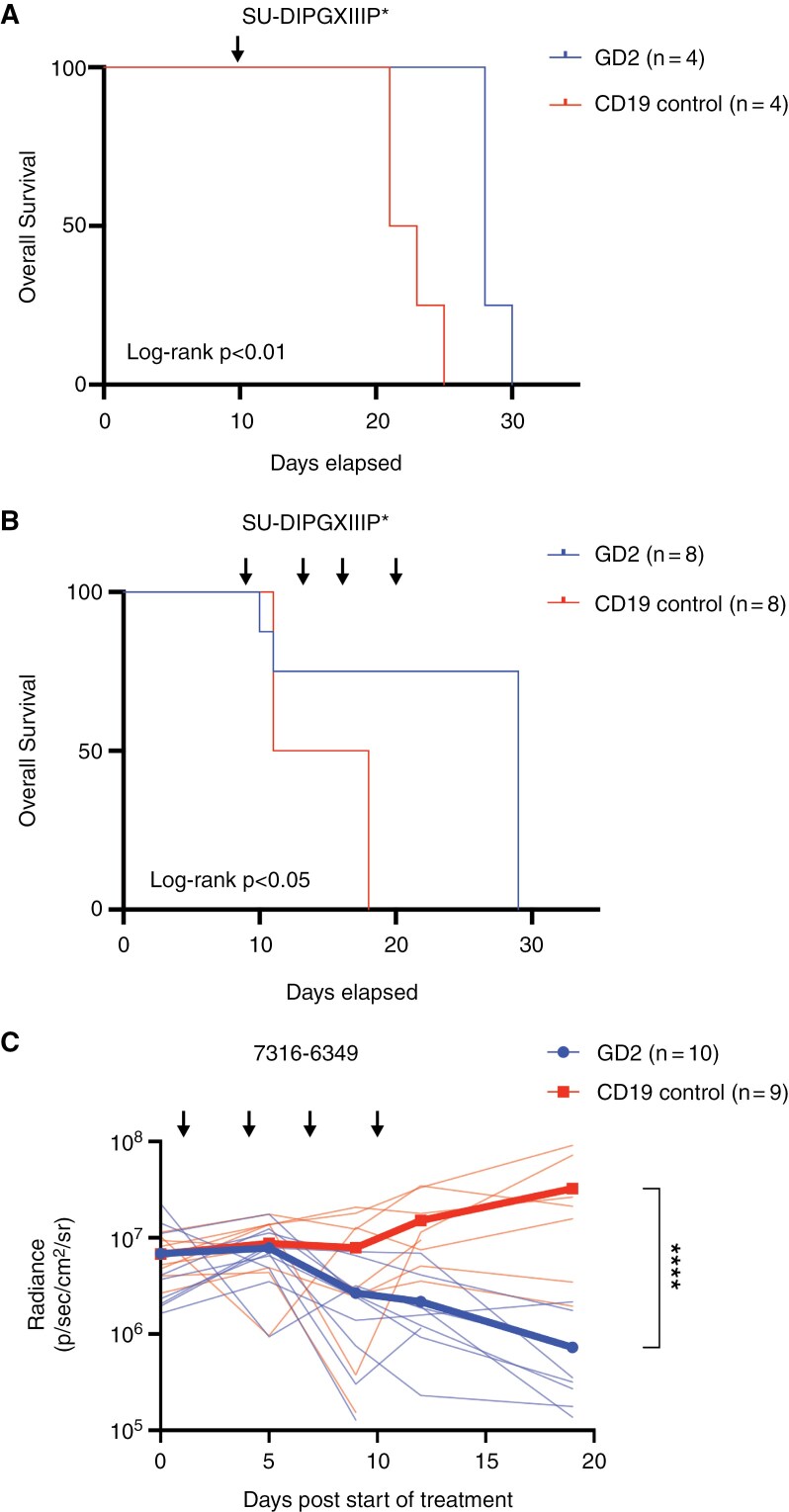
mRNA GD2 CAR T cells are active in pontine DMG murine models. (A) DIPGXIIIP* tumor cells were engrafted in the pons of NSG mice and treated with a single intratumoral dose of 2 × 10^6^ CAR T cells, either GD2-directed or CD19-directed control, indicated by the arrow. Survival shown as Kaplan–Meier. Significance calculated by log-rank test. (B) DIPGXIIIP* tumor cells were engrafted in the pons of NSG mice and treated with 4 intratumoral doses of 4 × 10^6^ CAR T cells, either GD2-directed or CD19-directed control, indicated by the arrows. Survival shown as Kaplan–Meier. Significance calculated by log-rank test. (C) 7316-6349 cells were engrafted in the pons of NSG mice and treated with 4 intratumoral doses of 4 × 10^6^ CAR T cells, either GD2-directed or CD19-directed control, indicated by the arrows. Tumor burden displayed as radiance and measured over time. Data shown as mean (thick line) and each individual animal (thin lines) for GD2-directed CAR treated animals in blue, and control mice in red. **** = *P* < .0001. Number of mice per group indicated in legends.

To evaluate our mRNA CAR T cells in murine models of DMG, we tested first in orthotopic xenografts of H3 K27M mutant cell lines injected orthotopically into the pons. As mRNA CAR T cells have a short half-life with limited time for trafficking, we treated with intratumoral dosing of CAR T cells as previously described.^29^ Prior data showed toxicity/fatality in a subset of mice with pontine SU-DIPGXIIIP* tumors treated with a systemic dose of viral GD2-directed CAR T cells,^4^ and so we first used a single intratumoral dose of mRNA CAR T cells to assess for toxicity. One intratumoral dose of 2 × 10^6^ mRNA GD2-directed CAR T cells did not show any toxic deaths after administration and improved median overall survival (OS) for mice from 22 days to 28 days compared to CD19-directed CAR controls (log-rank *P* < .01) (Figure 4A). Using the same model, we then increased the number of repeated intratumoral administrations to 4 doses of 4 × 10^6^ mRNA GD2-directed CAR T cells, or CD19-directed CAR T cells as controls, and OS improved to a median of 29 days compared to 14.5 days for controls (log-rank *P* < .05) (Figure 4B). To evaluate tumor burden using a bioluminescent model, we then treated mice engrafted with DMG model 7316-6349 injected into the pons with 4 repeated doses of 4 × 10^6^ mRNA GD2-directed CAR T cells or CD19-directed controls. Animals treated with GD2 CAR T cells showed decreased tumor burden at 3 weeks, with mean radiance of 7 × 10^5^ p/s/cm^2^/sr compared to 3 × 10^7^ p/s/cm^2^/sr in controls (*P* < .0001) (Figure 4C). As 7316-6349 tumor model is slower growing than SU-DIPGXIIIP*, mice succumbed to GVHD after 1 month from repeated T cell infusions prior to tumor-related death.

### mRNA GD2-Directed CAR T Cell Dosing Can Be Modulated to Improve Safety in Thalamic DMG

**Figure 5.**
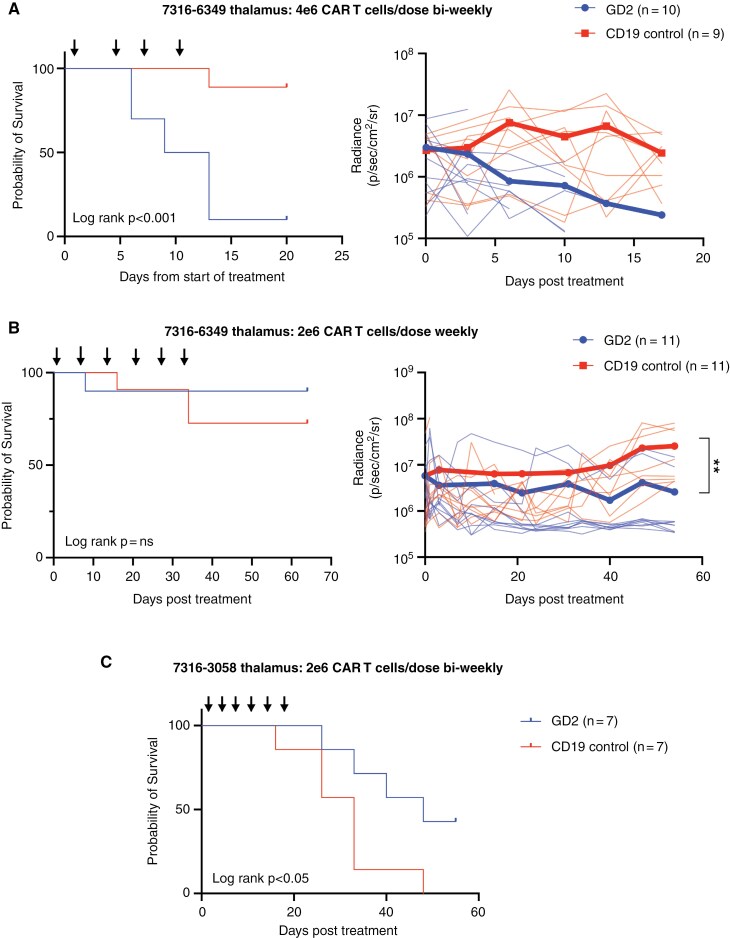
mRNA GD2 CAR T cells can be dose-adjusted to mitigate toxicity. (A) 7316-6349 tumor cells were engrafted in the thalamus of NSG mice and treated with 4 intratumoral doses of 4 × 10^6^ CAR T cells, either GD2-directed or CD19-directed control, given twice weekly indicated by the arrows. Survival shown as Kaplan–Meier on the left, tumor burden displayed as radiance and measured over time on the right. Tumor burden data shown as mean (thick line) and each individual animal (thin lines) for GD2-directed CAR treated animals in blue, and control mice in red. (B) 7316-6349 tumor cells were engrafted in the thalamus of NSG mice and treated with 6 intratumoral doses of 2 × 10^6^ CAR T cells, either GD2-directed or CD19-directed control, given once weekly indicated by the arrows. Survival shown as Kaplan–Meier on the left, tumor burden displayed as radiance and measured over time on the right. Tumor burden data shown as mean (thick line) and each individual animal (thin lines) for GD2-directed CAR treated animals in blue, and control mice in red. (C) 7316-3058 tumor cells engrafted in the thalamus of NSG mice and treated with 6 intratumoral doses of 2 × 10^6^ CAR T cells, either GD2-directed or CD19-directed control, given twice weekly indicated by the arrows. Survival shown as Kaplan–Meier, with significance calculated by log-rank test. ** = *P* < .01. Number of mice per group indicated in legends.

We next set out to evaluate mRNA CAR T cell efficacy and toxicity in thalamic models of DMG, where previous studies using viral GD2-directed CAR T cells showed significant toxicity and death of murine models by 14 days.^4^ Using 7316-6349 engrafted in the thalamus, we were able to recapitulate the toxicity from viral GD2-directed CAR T cells through aggressive intratumoral dosing of 4 × 10^6^ mRNA GD2-directed CAR T cells delivered twice weekly, where 9 of 10 animals died within 14 days of starting treatment despite decreasing tumor burden (median OS 11 days) (Figure 5A). GD2-directed CAR T cell-treated mice died abruptly, and their brains were not able to be collected for histologic analysis. The timeline of their death occurred more rapid than typically observed in this model, with radiance showing decrease in disease burden, suggesting death secondary to toxic complication rather than tumor. This was in contrast to control-treated mice where median OS was not reached (log-rank *P* < .001) (Figure 5A). We hypothesized that the transient nature of mRNA CAR T cells would allow for titration of cytotoxicity, thus enabling a narrow therapeutic window where mice with thalamic tumors could be treated effectively without toxic death. Treating the same model 7316-6349 engrafted in the thalamus with a lower dose of 2 × 10^6^ mRNA GD2-directed CAR T cells and spaced to weekly infusions showed improved survival of GD2-directed CAR T cell-treated mice with median OS not reached (Figure 5B). With the lower, spaced-out dose of T cells in this study, mice survived past 60 days before development of GVHD from the human T cells infused. Evaluation of bioluminescent imaging showed decrease tumor burden for GD2-directed CAR T treated mice compared to controls (mean radiance 2.6 × 10^6^ p/s/cm^2^/sr compared to 2.6 × 10^7^ p/s/cm^2^/sr, *P* < .01) (Figure 5B). To confirm our findings of decreased toxicity, we then evaluated a second thalamic DMG model 7316-3058. This model is aggressive and does not maintain luciferase, similar to pontine model SU-DIPGXIIIP*, but allows for evaluation of OS due to its aggressive growth in vivo. Repeated dosing of GD2-directed CAR T cells at 2 × 10^6^ mRNA T cells per dose given bi-weekly for a total of 6 doses resulted in improved overall survival (median OS 48 days versus 33 days, log-rank *P* < .05) (Figure 5C) without toxic deaths.

## Discussion

Use of mRNA as a therapeutic has gained enthusiasm since its recent success in developing the Sars-CoV2 vaccine.^32^ In this work, we show that mRNA can be electroporated into human T cells to produce CARs with effective cytotoxic killing against pediatric DMGs. These mRNA CAR T cells showed effective tumor killing in both cell lines and organoid models. Moreover, we take advantage of the transient properties of mRNA to provide titrated dosing that avoids in vivo toxicity in the pons and thalamus, which was previously reported when using viral GD2-directed CAR T cells. Viral CAR T cells are permanently altered to express their CAR and unfettered in their activation and expansion, while mRNA is finite and degraded over 7 to 10 days so that CAR activity is only present in prescribed amounts. Through smaller, less frequent dosing, we show that mRNA CAR T cells can thread the needle between efficacy and toxicity to provide decreased tumor burden and improved outcomes for thalamic DMG models.

Monje et al. recently published results from the first study arm treating patients with pontine and spinal DMG using lentiviral GD2-directed CAR T cells, where 4 patients experienced significant volumetric reductions in tumor size, and most showed at least transient improvement in neurologic deficits.^10^ This incredible achievement highlights the importance of continued refinement in targeting GD2, given its promising early efficacy. Of note, the trial reported dose-limiting toxicity with intravenous delivery of CAR T cells, and 100% of patients experienced tumor inflammation-associated neurotoxicity (TIAN) with intracerebroventricular (ICV) dosing, requiring intensive monitoring.^10^ Our mRNA may offer a less toxic alternative approach, especially for patients with thalamic primaries or more extensive tumor burden. While much of our work has focused on pontine and thalamic DMG, flow cytometry revealed elevated GD2 surface expression in other pHGGs, including histone wild-type tumors. This suggests our mRNA GD2-directed CAR T cells will likely be applicable across a broader cohort of HGGs in pediatric patients. In addition, the increase of GD2 in the H3 G34R recurrent sample may suggest treatment upregulates GD2 and allow us to tailor treatment sequence to optimize highest GD2 expression before treatment with CAR T cells. Future work will explore radiotherapy and other treatment options that may provide such antigen herding.

A limitation of our in vivo work is the intratumoral dosing, which was selected as it allows for a sensitive readout of our transient mRNA CAR T cells but is not the most common route of delivery currently used in the clinic. Most early phase trials for children with CNS tumors have been using intravenous (IV) or ICV routes of delivery. However, those options are not exclusive as Seattle Children’s BrainChild-01, -02, -03 (NCT03500991, NCT03638167, and NCT04185038, respectively) and City of Hope (NCT02208362) have all incorporated trial arms with intratumoral delivery.^7,33,34^ The majority of this experience is yet to be published, although a preliminary report does include a description of the tolerable intratumoral delivery of HER2 CAR T cells to a patient with HGG.^33^ Repeated MRI-guided stereotactic dosing has been used successfully for oncolytic virus, although often spaced out by 1 to several weeks between doses.^35^ Pontine DMG has been treated with a single intratumoral dose of oncolyctic virus,^36^ with plans to expand to a phase 2 trial with repeated intratumoral dosing. Indwelling skull mounted convection enhanced delivery (CED) devices have also been used in trials outside of CAR T cell therapy to successfully provide repeated intra-parenchymal/tumoral drug delivery,^37,38^ including trials for patients with pontine DMG^39^ and glioblastoma multiforme^40^ where repeated dosing through indwelling CED every 2 to 7 days was feasible and well tolerated. However, this approach would be novel for CAR T cell therapy and may not be feasible. Future work will be focused on evaluating alternative dosing strategies in the CNS, to assess if mRNA CAR T cells can be dosed into the CSF with similar efficacy and lack of toxicity.

In addition, our mRNA CAR T cells require repeated dosing for effect. While this has been standard practice in recent viral CNS CAR T cell clinical trials,^7,8,33^ the need for additional CAR T cells may limit the total number of doses available and thus hamper ultimate efficacy in human patients. Finally, in a prior trial using mRNA CAR T cells targeting mesothelin, the CAR provoked an anaphylaxis response in a patient after a prolonged break of 49 days between doses.^41^ Subsequently, anaphylaxis on the trial was prevented by ensuring all doses were delivered no longer than 10 days apart, thus it will be important to ensure similar timing in translation of the mRNA GD2-directed CAR T cell strategy.

As the initial proof-of-concept cases have emerged showing GD2-directed CAR T cells are active against neuroblastoma^6^ and DMG,^8^ it is imperative we optimize safety with this therapy in particular in the CNS, where TIAN^12^ can have fatal consequences. In addition, virally created CAR T cells offer risk of T cell mutagenesis with potential for T cell secondary malignancy, which has been reported in BCMA- and CD19-directed CAR T cells and is currently under investigation by the FDA.^42^ Our work offers an alternative strategy to deliver non-integrating, controlled doses of GD2-directed CAR T cells, and the ability to titrate CAR T cells to effect. The utility of mRNA as a transient payload can similarly be utilized for protein expression beyond CARs and opens a new avenue for bolstering cellular therapeutics.

## Supplementary material

Supplementary material is available online at *Neuro-Oncology* (https://academic.oup.com/neuro-oncology).

noaf115_Supplementary_Figures_S1-S4_Table_S1

## Data Availability

All molecular data from this manuscript is publicly available through OpenPBTA19 and GTEX20.
