# Supplementary material for: Transient mRNA CAR T cells targeting GD2 provide dose-adjusted efficacy against diffuse midline glioma and high-grade glioma models
Source: Neuro Oncol. 2025 May 24;27(10):2684–96. doi: 10.1093/neuonc/noaf115 (PMC12833531; doi:10.1093/neuonc/noaf115)
Supplement: noaf115_Supplementary_Figures_S1-S4_Table_S1 [file noaf115_supplementary_figures_s1-s4_table_s1.docx]

| **Supplemental Figure 1** |  |
| --- | --- |
|  |  |
|  |  |


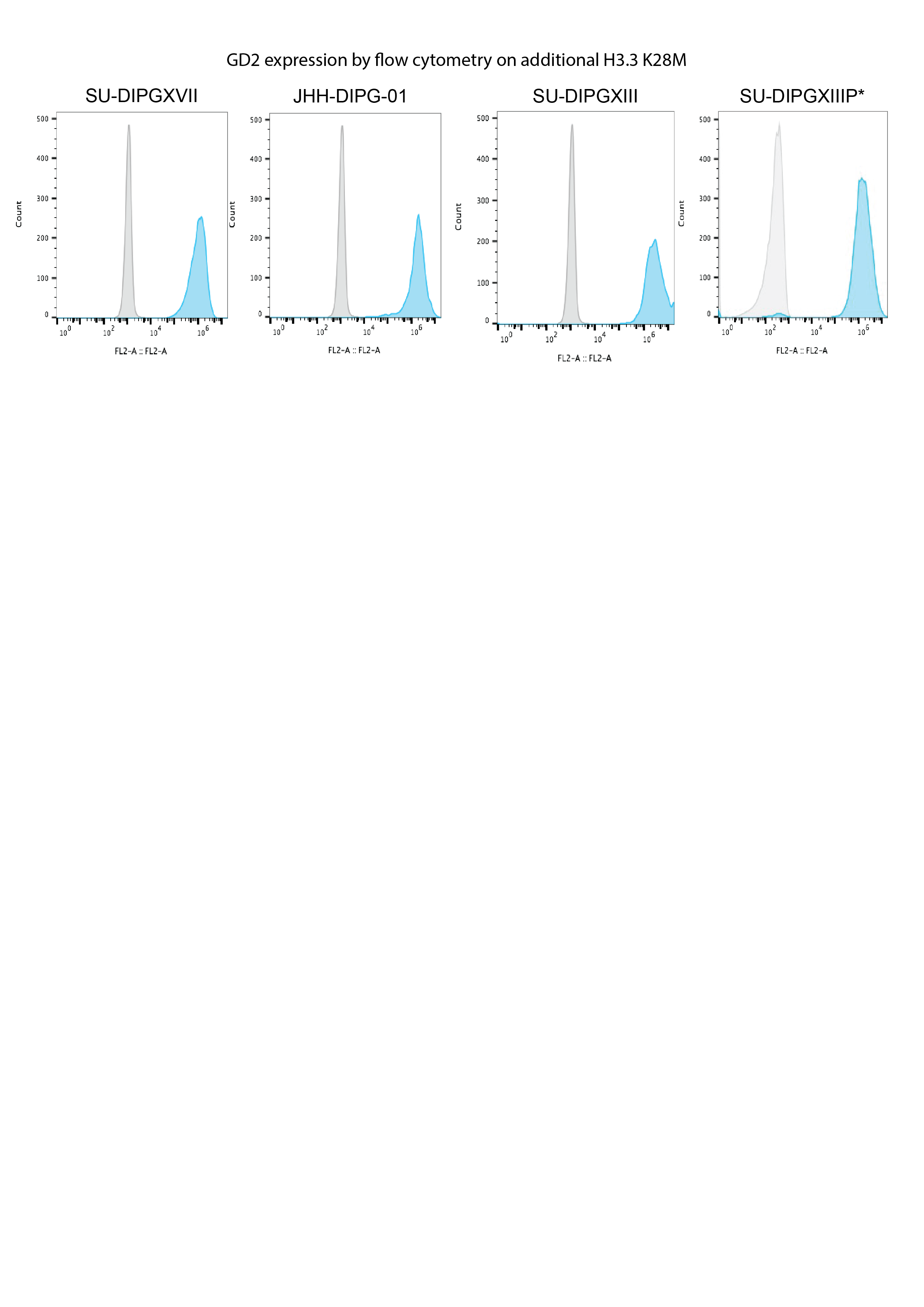


Supplemental Figure 1: Flow cytometry of H3.3 K27M additional cell lines. Control cell line shown in gray, tumor cell line in blue. Histogram displaying fluorescence intensity.

**Supplemental Figure 2
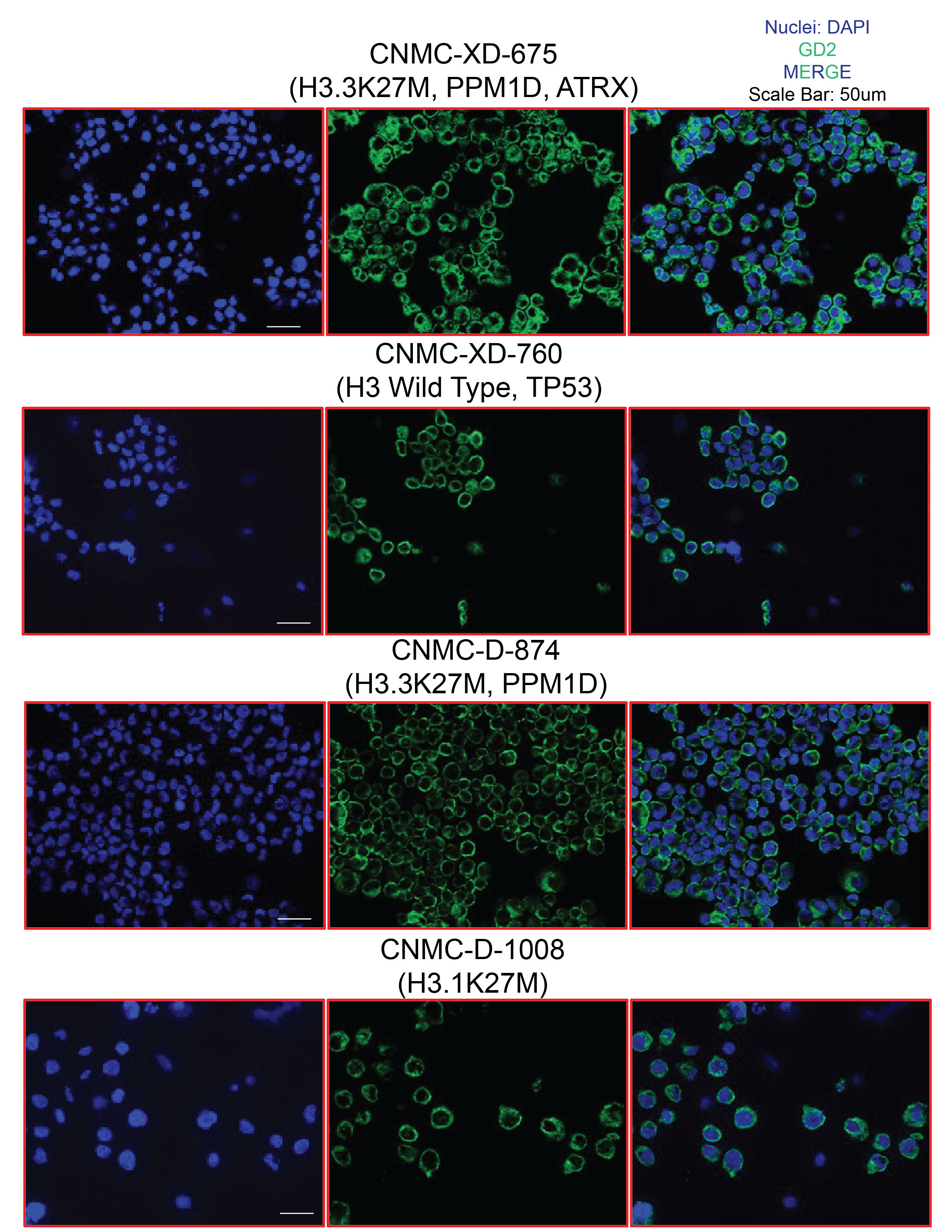
**

Supplemental Figure 2: Immunofluorescence of H3 wild type and mutant cell lines. Blue indicates Dapi stain, green indicates GD2 stain. Scale bar is 50 micrometers.

**Supplemental Figure 3**

Supplemental figure 3: LASSO, Ridge, and Elastic-net penalized linear regression modeling to integrate mutational, copy-number, and RNA-level data modalities from 175 DMG patients from OpenPBTA v12. a) Alpha optimization plot running ten-fold penalized linear regression of mutational, copy-number and transcriptomic profiles to identify alpha value with minimized cross-validation error. b) Lasso regression mean-squared error (MSE) of penalized fit at different regularization penalty (lambda) values. Values on top of plot indicate number of variables that are included as lambda increases. c) Ridge regression mean-squared error (MSE) of penalized fit at different regularization penalty (lambda) values. Values on top of plot indicate number of variables that are included as lambda increases. d) Elastic-net regression mean-squared error (MSE) of penalized fit at different regularization penalty (lambda) values using optimal alpha value from (a). Values on top of plot indicate number of variables that are included as lambda increases.

**Supplemental Figure 4**

**
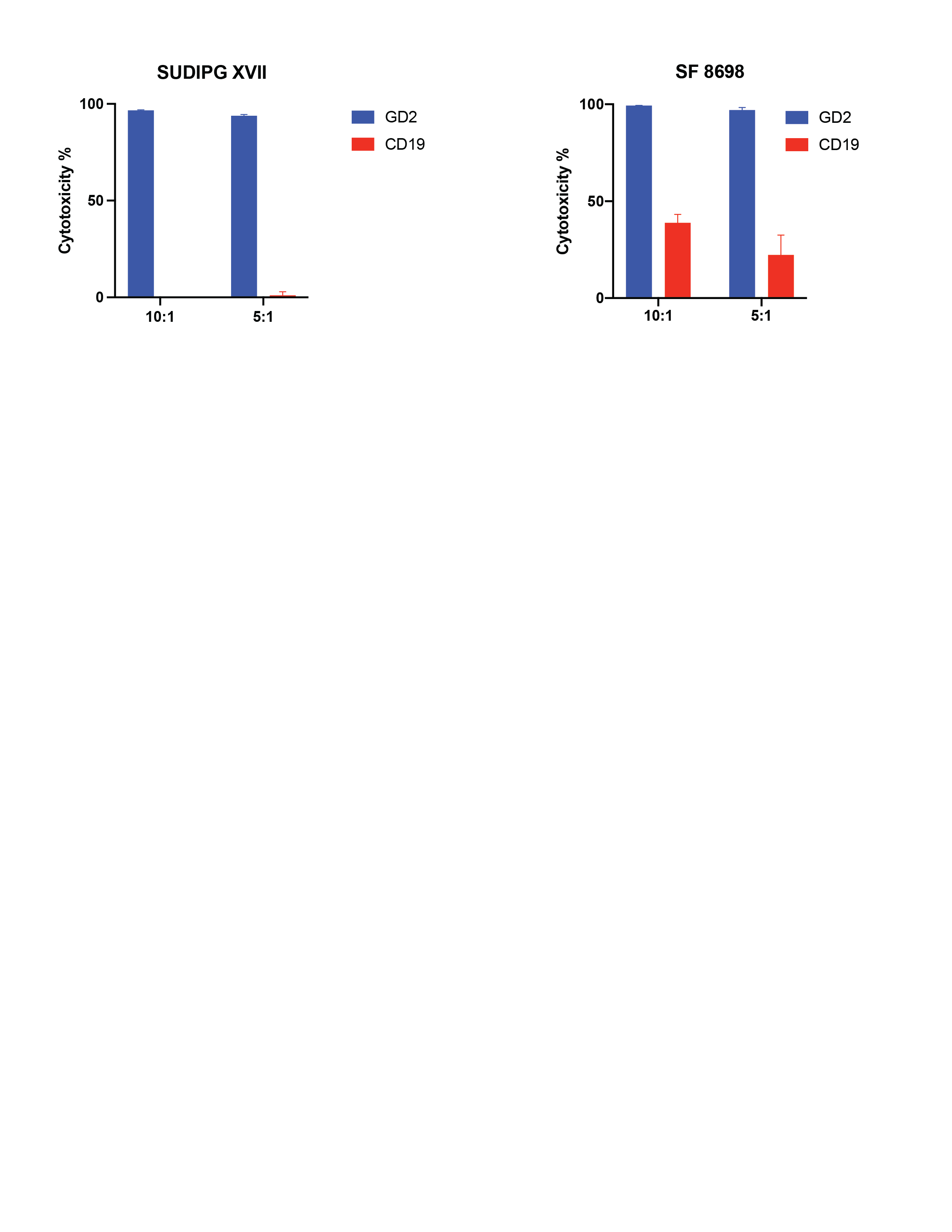
**

Supplemental Figure 3: *In vitro* cytotoxicity of GD2-directed CAR T cells. Cytotoxicity determined using luciferase assay after 48 hours of co-incubation. GD2-directed CAR T cells in blue, control CD19-directed CAR T cells in red, each shown at E:T ratios of 10:1 and 5:1.

**Supplemental Table 1**

| **Original sequence** | AUGGAGUUCGGGCUGAGUUGGCUGUUCCUGGUGGCUAUCCUGAAGGGCGUGCAGUGCUCUCGGGACAUCCUGCUGACCCAGACACCCCUGUCCCUGCCAGUGUCUCUGGGCGACCAGGCCUCUAUCAGCUGCCGCAGCUCCCAGUCUCUGGUGCACCGGAACGGCAAUACCUACCUGCACUGGUAUCUGCAGAAGCCCGGCCAGAGCCCUAAGCUGCUGAUCCACAAGGUGAGCAACAGAUUCUCCGGCGUGCCCGACAGGUUUUCCGGCUCUGGCAGCGGCACCGAUUUCACACUGAAGAUCUCCAGAGUGGAGGCCGAGGACCUGGGCGUGUACUUCUGCUCCCAGUCUACCCACGUGCCACCUCUGACCUUUGGCGCAGGAACAAAGCUGGAGCUGAAGAGGGCAGAUGCAGCACCAACAGUGUCUAUCUUUCCAGGAAGCGGAGGAGGAGGAUCCGGAGGAGAGGUGAAGCUGCAGCAGUCUGGACCUAGCCUGGUGGAGCCAGGAGCCUCUGUGAUGAUCAGCUGUAAGGCCUCCGGCUCUAGCUUCACCGGCUAUAACAUGAAUUGGGUGCGCCAGAACAUCGGCAAGAGCCUGGAGUGGAUCGGCGCCAUCGACCCUUACUAUGGCGGCACAUCCUACAAUCAGAAGUUUAAGGGCCGGGCCACCCUGACAGUGGACAAGUCCUCUAGCACCGCCUAUAUGCACCUGAAGUCCCUGACAUCUGAGGAUAGCGCCGUGUACUAUUGCGUGUCUGGCAUGGAGUACUGGGGCCAGGGCACCAGCGUGACAGUGUCCUCUGCCAAGACCACACCACCCUCCGUGUAUGGAAGGGUGACCGUGAGCUCCGCCGAGCCAAAGUCCUGUGAUAAGACCCACACAUGCCCUCCAUGUCCUGGCUCUAUCUACAUCUGGGCACCACUGGCAGGAACCUGCGGCGUGCUGCUGCUGUCCCUGGUCAUCACCCUGUACUGUAAGAGAGGCAGGAAGAAGCUGCUGUAUAUCUUCAAGCAGCCCUUCAUGCGGCCCGUGCAGACCACACAGGAGGAGGACGGCUGCAGCUGUAGGUUCCCAGAAGAGGAGGAGGGAGGAUGUGAGCUGCGCGUGAAGUUUAGCCGGUCCGCCGAUGCACCUGCAUACCAGCAGGGCCAGAAUCAGCUGUAUAACGAGCUGAAUCUGGGCCGGAGAGAGGAGUACGACGUGCUGGAUAAGAGGAGGGGACGGGAUCCCGAGAUGGGAGGCAAGCCCCGGAGAAAGAACCCUCAGGAGGGCCUGUACAAUGAGCUGCAGAAGGACAAGAUGGCCGAGGCCUAUAGCGAGAUCGGCAUGAAGGGAGAGAGGCGCCGGGGCAAGGGACACGAUGGCCUGUAUCAGGGCCUGUCCACCGCUACCAAGGAUACCUAUGAUGCUCUGCACAUGCAGGCUCUGCCCCCCCGA |
| --- | --- |
| **Frameshift 1** | AUGGAGUUCGGGCUGAGUUGGCUGUUCCUGGUGGCUAUCCUGAAGGGCGUGCAGUGCUCUCGGGACAUCCUGCUGACCCAGACACCCCUGUCCCUGCCAGUGUCUCUGGGCGACCAGGCCUCUAUCAGCUGCCGCAGCUCCCAGUCUCUGGUGCACCGGAACGGCAAUACCUACCUGCACUGGUAUCUGCAGAAGCCCGGCCAGAGCCCUAAGCUGCUGAUCCACAAGGUGAGCAACAGAUUCUCCGGCGUGCCCGACAGGUUUCCGGCUCUGGCAGCGGCACCGAUUUCACACUGAAGAUCUCCAGAGUGGAGGCCGAGGACCUGGGCGUGUACUUCUGCUCCCAGUCUACCCACGUGCCACCUCUGACCUUUGGCGCAGGAACAAAGCUGGAGCUGAAGAGGGCAGAUGCAGCACCAACAGUGUCUAUCUUUCCAGGAAGCGGAGGAGGAGGAUCCGGAGGAGAGGUGAAGCUGCAGCAGUCUGGACCUAGCCUGGUGGAGCCAGGAGCCUCUGUGAUGAUCAGCUGUAAGGCCUCCGGCUCUAGCUUCACCGGCUAUAACAUGAAUUGGGUGCGCCAGAACAUCGGCAAGAGCCUGGAGUGGAUCGGCGCCAUCGACCCUUACUAUGGCGGCACAUCCUACAAUCAGAAGUUUAAGGGCCGGGCCACCCUGACAGUGGACAAGUCCUCUAGCACCGCCUAUAUGCACCUGAAGUCCCUGACAUCUGAGGAUAGCGCCGUGUACUAUUGCGUGUCUGGCAUGGAGUACUGGGGCCAGGGCACCAGCGUGACAGUGUCCUCUGCCAAGACCACACCACCCUCCGUGUAUGGAAGGGUGACCGUGAGCUCCGCCGAGCCAAAGUCCUGUGAUAAGACCCACACAUGCCCUCCAUGUCCUGGCUCUAUCUACAUCUGGGCACCACUGGCAGGAACCUGCGGCGUGCUGCUGCUGUCCCUGGUCAUCACCCUGUACUGUAAGAGAGGCAGGAAGAAGCUGCUGUAUAUCUUCAAGCAGCCCUUCAUGCGGCCCGUGCAGACCACACAGGAGGAGGACGGCUGCAGCUGUAGGUUCCCAGAAGAGGAGGAGGGAGGAUGUGAGCUGCGCGUGAAGUUUAGCCGGUCCGCCGAUGCACCUGCAUACCAGCAGGGCCAGAAUCAGCUGUAUAACGAGCUGAAUCUGGGCCGGAGAGAGGAGUACGACGUGCUGGAUAAGAGGAGGGGACGGGAUCCCGAGAUGGGAGGCAAGCCCCGGAGAAAGAACCCUCAGGAGGGCCUGUACAAUGAGCUGCAGAAGGACAAGAUGGCCGAGGCCUAUAGCGAGAUCGGCAUGAAGGGAGAGAGGCGCCGGGGCAAGGGACACGAUGGCCUGUAUCAGGGCCUGUCCACCGCUACCAAGGAUACCUAUGAUGCUCUGCACAUGCAGGCUCUGCCCCCCCGA |
| **Frameshift 2** | AUGGAGUUCGGGCUGAGUUGGCUGUUCCUGGUGGCUAUCCUGAAGGGCGUGCAGUGCUCUCGGGACAUCCUGCUGACCCAGACACCCCUGUCCCUGCCAGUGUCUCUGGGCGACCAGGCCUCUAUCAGCUGCCGCAGCUCCCAGUCUCUGGUGCACCGGAACGGCAAUACCUACCUGCACUGGUAUCUGCAGAAGCCCGGCCAGAGCCCUAAGCUGCUGAUCCACAAGGUGAGCAACAGAUUCUCCGGCGUGCCCGACAGGUUUUCCGGCUCUGGCAGCGGCACCGAUUUCACACUGAAGAUCUCCAGAGUGGAGGCCGAGGACCUGGGCGUGUACUUCUGCUCCCAGUCUACCCACGUGCCACCUCUGACCUUUGGCGCAGGAACAAAGCUGGAGCUGAAGAGGGCAGAUGCAGCACCAACAGUGUCUAUCUUUCAGGAAGCGGAGGAGGAGGAUCCGGAGGAGAGGUGAAGCUGCAGCAGUCUGGACCUAGCCUGGUGGAGCCAGGAGCCUCUGUGAUGAUCAGCUGUAAGGCCUCCGGCUCUAGCUUCACCGGCUAUAACAUGAAUUGGGUGCGCCAGAACAUCGGCAAGAGCCUGGAGUGGAUCGGCGCCAUCGACCCUUACUAUGGCGGCACAUCCUACAAUCAGAAGUUUAAGGGCCGGGCCACCCUGACAGUGGACAAGUCCUCUAGCACCGCCUAUAUGCACCUGAAGUCCCUGACAUCUGAGGAUAGCGCCGUGUACUAUUGCGUGUCUGGCAUGGAGUACUGGGGCCAGGGCACCAGCGUGACAGUGUCCUCUGCCAAGACCACACCACCCUCCGUGUAUGGAAGGGUGACCGUGAGCUCCGCCGAGCCAAAGUCCUGUGAUAAGACCCACACAUGCCCUCCAUGUCCUGGCUCUAUCUACAUCUGGGCACCACUGGCAGGAACCUGCGGCGUGCUGCUGCUGUCCCUGGUCAUCACCCUGUACUGUAAGAGAGGCAGGAAGAAGCUGCUGUAUAUCUUCAAGCAGCCCUUCAUGCGGCCCGUGCAGACCACACAGGAGGAGGACGGCUGCAGCUGUAGGUUCCCAGAAGAGGAGGAGGGAGGAUGUGAGCUGCGCGUGAAGUUUAGCCGGUCCGCCGAUGCACCUGCAUACCAGCAGGGCCAGAAUCAGCUGUAUAACGAGCUGAAUCUGGGCCGGAGAGAGGAGUACGACGUGCUGGAUAAGAGGAGGGGACGGGAUCCCGAGAUGGGAGGCAAGCCCCGGAGAAAGAACCCUCAGGAGGGCCUGUACAAUGAGCUGCAGAAGGACAAGAUGGCCGAGGCCUAUAGCGAGAUCGGCAUGAAGGGAGAGAGGCGCCGGGGCAAGGGACACGAUGGCCUGUAUCAGGGCCUGUCCACCGCUACCAAGGAUACCUAUGAUGCUCUGCACAUGCAGGCUCUGCCCCCCCGA |

Supplemental table 1: Alternative open reading frames (ORFs) identified from potential mRNA frame-shifting.
